# Gas Sensing Analysis of Ag-Decorated Graphene for Sulfur Hexafluoride Decomposition Products Based on the Density Functional Theory

**DOI:** 10.3390/s16111830

**Published:** 2016-11-01

**Authors:** Xiaoxing Zhang, Rong Huang, Yingang Gui, Hong Zeng

**Affiliations:** 1 State Key Laboratory of Power Transmission Equipment & System Security and New Technology, Chongqing University, Chongqing 400044, China; 20141113077@cqu.edu.cn (R.H.); yingang.gui@gmail.com (Y.G.); 2 School of Electrical Engineering, Wuhan University, Wuhan 430072, China; 3 No. 1 Branch of Chongqing Academy of Metrology and Quality Inspection, Chongqing 402260, China; zhabc66@163.com

**Keywords:** density functional theory, sulfur hexafluoride decomposition product, Ag-graphene, gas sensor

## Abstract

Detection of decomposition products of sulfur hexafluoride (SF_6_) is one of the best ways to diagnose early latent insulation faults in gas-insulated equipment, and the occurrence of sudden accidents can be avoided effectively by finding early latent faults. Recently, functionalized graphene, a kind of gas sensing material, has been reported to show good application prospects in the gas sensor field. Therefore, calculations were performed to analyze the gas sensing properties of intrinsic graphene (Int-graphene) and functionalized graphene-based material, Ag-decorated graphene (Ag-graphene), for decomposition products of SF_6_, including SO_2_F_2_, SOF_2_, and SO_2_, based on density functional theory (DFT). We thoroughly investigated a series of parameters presenting gas-sensing properties of adsorbing process about gas molecule (SO_2_F_2_, SOF_2_, SO_2_) and double gas molecules (2SO_2_F_2_, 2SOF_2_, 2SO_2_) on Ag-graphene, including adsorption energy, net charge transfer, electronic state density, and the highest and lowest unoccupied molecular orbital. The results showed that the Ag atom significantly enhances the electrochemical reactivity of graphene, reflected in the change of conductivity during the adsorption process. SO_2_F_2_ and SO_2_ gas molecules on Ag-graphene presented chemisorption, and the adsorption strength was SO_2_F_2_ > SO_2_, while SOF_2_ absorption on Ag-graphene was physical adsorption. Thus, we concluded that Ag-graphene showed good selectivity and high sensitivity to SO_2_F_2_. The results can provide a helpful guide in exploring Ag-graphene material in experiments for monitoring the insulation status of SF_6_-insulated equipment based on detecting decomposition products of SF_6_.

## 1. Introduction

Sulfur hexafluoride (SF_6_) has been widely applied in gas-insulated switchgears (GIS) because of its excellent insulation and arc-quenching performance [1]. When the insulation performance of electrical equipment begins to decline, the energy released by the partial discharge phenomena will cause SF_6_ to decompose into multiple low fluorine sulfides [2]. These low fluorine sulfides react with trace water and oxygen to generate SO_2_F_2_, SOF_2_, SO_2_, and other characteristic gas products in GIS [3]. Based on previous research, decomposition and concentration of gas products are closely associated with the type, severity, and generation factor of partial discharge [4]. Therefore, detection of SF_6_ decomposition products is an effective way to monitor and diagnose insulation faults in gas-insulated electrical equipment.

The broad application prospect of graphene is due to its unique structure, electronic properties, and excellent electrical performance [5,6,7,8]. Moreover, graphene possesses many advantages, such as an extremely large specific surface area [9], great carrier concentration [10], ultrahigh carrier mobility [10], low Johnson noise, fewer internal defects [11], and zero-gap materials (with metallicity) [12]. In 2007, Schedin first researched adsorption and desorption behavior of single gas molecules on intrinsic graphene surfaces and expanded the research of graphene as a gas sensor [13]. Researchers confirmed that graphene with certain defects or decorations had stronger response characteristics to specific gases, and the selectivity and sensitivity of graphene to a particular gas can be effectively improved through conducting specific doping or functional modification [14]. At present, the research on functionalized graphene is limited to a common detection field, such as NO, H_2_, and other common gases [15,16]. Meanwhile, a few research reports have been written about its application in detection of SF_6_ decomposition products. According to the preliminary study in our study group and its high hydrogen uptake in a previous paper, Ag-graphene gas sensor has good gas-sensing properties for SF_6_ decomposition products [17,18,19]. In addition, feasible fabrication of Ag-graphene will lead to large-scale industrial production [20]. To further guide experiments to enhance the detection sensitivity and selectivity of Ag-graphene gas sensors for SF_6_ decomposition products, the gas-sensing properties and gas-response mechanism of Ag-graphene sensors for different SF_6_ decomposition products must be further analyzed.

Based on density functional theory (DFT), adsorption processes of single (SO_2_F_2_, SOF_2_ and SO_2_) and double gas molecules (2SO_2_F_2_, 2SOF_2_, 2SO_2_) on Ag-graphene were analyzed, respectively. The theory suggested that decorated Ag could effectively improve the electron density and charge transfer characteristics of graphene, which provided a new way of preparing sensing materials for functionalized graphene gas and developing a high-performance gas sensor for detection of SF_6_ decomposition products.

## 2. Materials and Methods

All calculations were based on DMol^3^ module of Materials Studio. First, we chose a 6 × 6 graphene supercell (72 carbon atoms) to build the graphene structure, and the periodic boundary condition of supercell was set to 20 Å to avoid the interaction between adjacent layers in modeling. Then, optimization calculation was conducted for the gas molecule structure and the supercell to adjust their structure parameters in the model to be as close to the experimental parameters as possible. To find the most stable Int-graphene and Ag-graphene structure after adsorbing gas molecules, the system of gas molecules on Int-graphene and Ag-graphene was calculated and optimized. Finally, obtained adsorption systems were analyzed by a series of physical and chemical characteristic parameters. Because the Van der Waals force shows obvious effects in physical adsorption, this work selected the generalized gradient approximation method, using Perdew-Burke-Ernzehof format and dispersion correction for DFT algorithm to process exchange correlation and iteration process, respectively [21]. Thus, the influence of the Van der Waals force on adsorption effect could be explored more accurately. In addition, a basis set of P-polarized double-number orbit was added; a *k*-point mesh of 6 × 6 × 1 was selected for the Brillouin zone integration to obtain accurate energies and structures; and the convergence tolerance of energy was 2.72 × 10^−5^ eV in structural optimization [22]. Previously published research agreed with the model and its related parameters established in this work [23]. 

After presenting the optimization structure, a series of physical and chemical parameters were analyzed to understand the adsorption process of gas molecules on Int-graphene and Ag-graphene. Their definitions are as follows:

Adsorption distance (d) means the closest distance from Int-graphene or Ag-graphene to gas molecules. Adsorption energy (∆E_ads_), the acting force of gas molecules adsorbed on Int-graphene and Ag-graphene, gives the change of total energy in the adsorption process of gas molecules on Int-graphene and Ag-graphene to reach the most stable adsorption system using the equation:

ΔE_ads_ = E_(system)_ − E_(Int-graphene/Ag-graphene)_ − E_(molecule)_, (1)

where E_(system)_, E_(Int-graphene/Ag-graphene)_, and E_(molecule)_ are the total energy of adsorption system after the molecules adsorbed on graphene or Ag-graphene, the energy of graphene or Ag-graphene, and the energy of gas molecules, respectively.

Net charge transfer (Q) characterizes the quantity of electric charge transferred from gas molecules to Int-graphene or Ag-graphene in the absorption process and reflects the change of electrical properties of Int-graphene and Ag-graphene. The energy gap of adsorption system offers the complexity of electron transfer between the whole highest occupied molecular orbital (HOMO) and lowest unoccupied molecular orbital (LUMO), and its formula is as follows:

E_g_ = |E_HOMO_ − E_LUMO_| (2)

## 3. Results

### 3.1. Establishment of the Geometric Model

**Figure 1:**
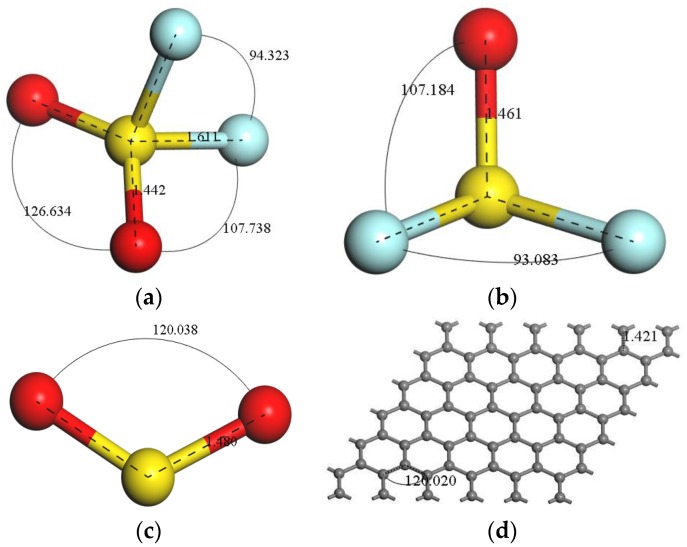
Geometric structures after optimization in (**a**) SO_2_F_2_ molecule; (**b**) SOF_2_ molecule; and (**c**) SO_2_ molecule and (**d**) Int-graphene.

To find the steadiest state, gas molecule models of SF_6_ characteristic decomposition (SO_2_F_2_, SOF_2_ and SO_2_) and Int-graphene were constructed in material visualizer. Then, every geometrical structure was optimized by finding its lowest energy, so bond length and angle were marked in each optimized gas molecule model (see Figure 1), whose parameters were consistent with the published data from other simulation results [24]. 

**Figure 2:**
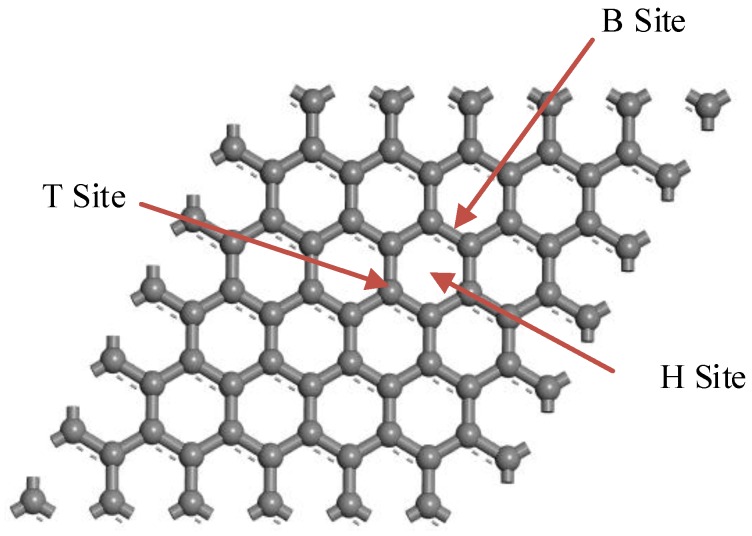
Three possible doping sites for an Ag atom.

We explored the decoration mechanism of a single Ag atom on graphene. After optimizing the geometrical structure of graphene, one Ag atom was embedded on the graphene plane in each unit cell. To reach the most stable decorated graphene with a single atom, three possible positions were considered according to the characteristics of highly symmetrical structure of graphene and the previous literature. Figure 2 shows the top site directly above a C atom (T-site), the hollow site at the center of a hexagon (H-site), and the bridge site at the midpoint of two C atoms (B-site).

The binding energy describes the natural interaction of the bonding between decorated Ag and graphene as:

E_form_ = −E(C_72_) − E(Ag) + E(Ag/C_72_), (3)

where E (C_72_), E (Ag), and E (Ag/C_72_) represent the respective energy of intrinsic graphene, energy of a single Ag atom, and the total energy of optimized Ag-graphene.

Table 1 shows details of the binding energy in three possible positions, demonstrating that T-site holds the minimum energy. Moreover, the T-site model could be speculated to be the main decorated structure during experimental preparation according to the single metal atom decorated in the same method [25]. Therefore, we regarded the T-site model as the gas-sensing material to study its gas sensing properties to SF_6_ decomposition products, which was verified and used in previous similar studies [26].

**Figure 3:**
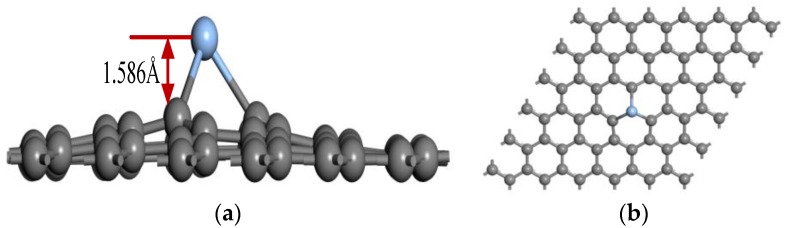
Partial atomic structure of (**a**) side view and (**b**) top view for the Ag-C_T_ doping graphene surface.

**Figure 4:**
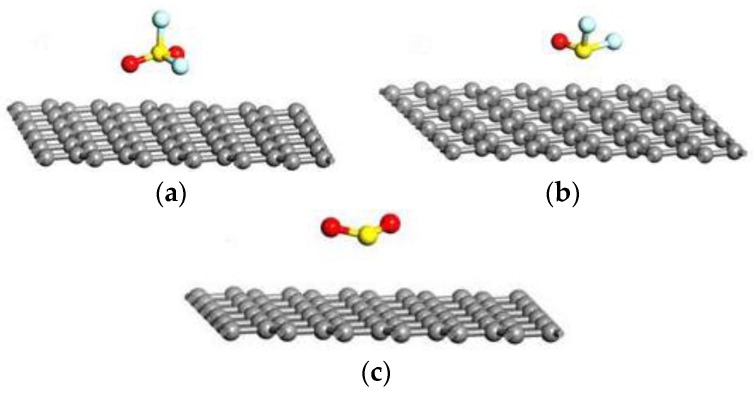
The most stable optimized geometric of single gas molecules interacting with Int-graphene in (**a**) Int-graphene/SO_2_F_2_; (**b**) Int-graphene/SOF_2_; and (**c**) Int-graphene/SO_2_.

Figure 3a and Figure 4b respectively offer the side and top views of the T-site model after geometry optimization, which illustrates that the Ag atom embedded on the graphene does not lead to the change of the whole two-dimensional plane structure of graphene, but rather protrudes out of the C atomic layer plane with distance (1.586 Å) along the *Z*-direction and forms covalent bonds with its adjacent C atoms with 2.053 Å bond length. This further supports the idea that an Ag-decorated graphene structure has a local sp^3^ configuration. Otherwise, the similar decorated structures of local sp^3^ configuration were also found in other metal-element-decorated graphene (Pd, Pt, Mn, etc.), and it is generally believed that local sp^3^ configuration is correct in metal-doped graphene theory research [27].

### 3.2. Adsorption of a Single Gas Molecule on Int-Graphene

To have a better understanding of adsorption effects of gas molecules on Ag-graphene, we first investigated single gas molecule (SO_2_F_2_, SOF_2_, and SO_2_) adsorption with different initial positions. We focused on a series of parameters presenting gas-sensing properties of adsorption process, such as d, ∆E_ads_, and Q. Figure 4 shows the optimized stable adsorption systems holding the lowest energy among similar configurations, and Table 2 shows their parameters.

As shown in Figure 4 and Table 2, graphene interacted weakly with gas molecules, and the structure of these molecules almost remains the same (see in Figure 1). The E_ads_ and d were −0.463 eV and 3.486 Å for SO_2_F_2_, respectively. ∆E_ads_ and d of graphene/SO_2_ were −0.459 eV and 3.531 Å. The value of ∆E_ads_ and d upon SO_2_ adsorption on graphene were −0.298 eV and 3.344 Å, respectively. In addition, net charge transfers (Q) were −0.004 e, −0.002 e and −0.003 e in graphene/SO_2_F_2_, graphene/SOF_2_ and graphene/SO_2_, respectively. Consequently, the interaction for gas molecule adsorption on graphene is just van der Waals interactions, which are too weak to influence the change of conductivity in the gas adsorption process. Therefore, intrinsic graphene material is not suitable for decomposition component detection in SF_6_-insulated equipment. Thus, we do not further discuss the adsorption details for gas molecules adsorption on intrinsic graphene.

### 3.3. Adsorption of a Single Gas Molecule on Ag-Graphene

**Figure 5:**
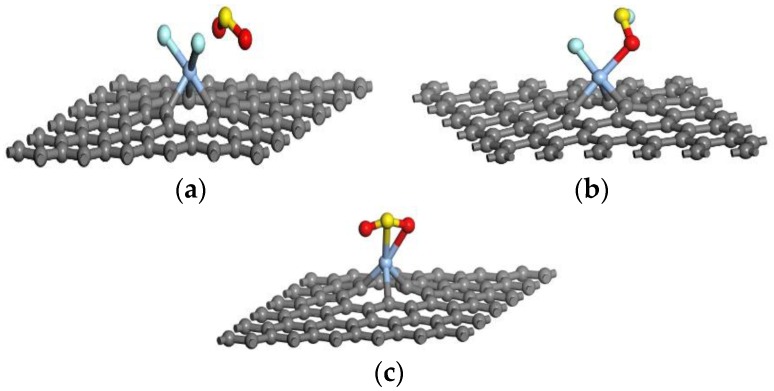
The most stable optimized geometric of single gas molecules interacting with Ag-graphene in (**a**) Ag-graphene/SO_2_F_2_; (**b**) Ag-graphene/SOF_2_; and (**c**) Ag-graphene/SO_2_.

To obtain the most stable adsorption system, single gas molecules (SO_2_F_2_, SOF_2_ and SO_2_) were made closer to Ag-graphene with different initial positions. We computed same parameters to analyze the gas-sensing properties of the adsorbing process. These optimized stable adsorption systems are exhibited in Figure 5, and all their parameters are shown in Table 3.

As shown in Figure 5 and Table 3, Ag-graphene and SO_2_F_2_ have a strong interaction. Two F atoms separated from SO_2_F_2_ and approached decorated Ag with two S-F bonds of SO_2_F_2_, extending to 1.948 Å and 3.942 Å, respectively, in the adsorption process, which indicates SO_2_F_2_ decomposition. Meanwhile, the value of Q upon SO_2_F_2_ adsorption on Ag-graphene was −0.947 e, which meant that SO_2_F_2_ acted as an electron acceptor during electron transfer, because electron-rich F atoms contributed to pulling electrons from Ag-graphene to SO_2_F_2_. Another essential parameter ∆E_ads_ verified that the strong interaction belonged to chemisorption, because ∆E_ads_ of SO_2_F_2_ on Ag-graphene (−1.448 eV) had far exceeded the critical value of chemical adsorption (0.8 eV) [26].

However, a different case happened with SOF_2_. Figure 5b displays the most stable adsorption system, and its related data is shown in Table 3. When SOF_2_ came close to Ag-graphene, the F atom tried to separate from SOF_2_, so the S-F bond was stretched to 2.718 Å, which offered a chance to build a new bond between Ag interacted, and lone F and left S atoms, respectively. However, these interactions were some kind of physical adsorption because of low Van der Waals force with bonding energy (−0.678 eV). In addition, the value of Q upon SOF_2_ adsorption on Ag-graphene was only −0.351 e. This agrees with the conclusion about physical adsorption, which indicates that electron also transfers from Ag-graphene to gas molecule. 

SO_2_ adsorption did not show obvious changes on molecular gas structure (see Figure 5). While the bond length of S-O in SO_2_ molecule had extended from 1.480 Å to 1.572 Å, distances from O and S atoms of SO_2_ to Ag atom gradually shortened to 2.210 Å and 2.558 Å, respectively. SO_2_-Ag-graphene interaction formed new Ag-S and Ag-O covalent bonds, as shown in Figure 5, thus leading to S-O bond stretching in SO_2_ molecule. Meanwhile, ∆E_ads_ of Ag-graphene/SO_2_ (−1.075 eV) was lower than that of Ag-graphene/SO_2_F_2_, but higher than that of Ag-graphene/SOF_2_. Similarly, the value of Q (−0.801 e) upon SO_2_ adsorption on Ag-graphene was the middle value among three cases. Therefore, SO_2_ exhibited chemisorption with Ag-graphene.

Net charge transfer has an effect on the density of states (DOS), which results in the change of conductance in system. Thus, the density of states (DOS) and its corresponding partial DOS (PDOS) of Ag-graphene before and after adsorption were calculated to further analyze the chemical adsorption mechanism of gas on Ag-graphene.

**Figure 6:**
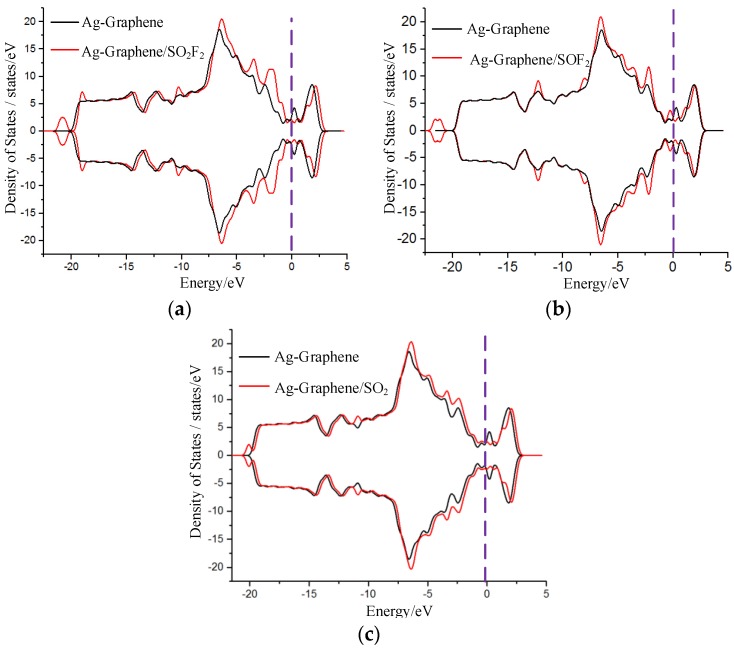
DOS for Ag-graphene with and without single-molecule adsorption in (**a**) SO_2_F_2_; (**b**) SOF_2_; and (**c**) SO_2_.

**Figure 7:**
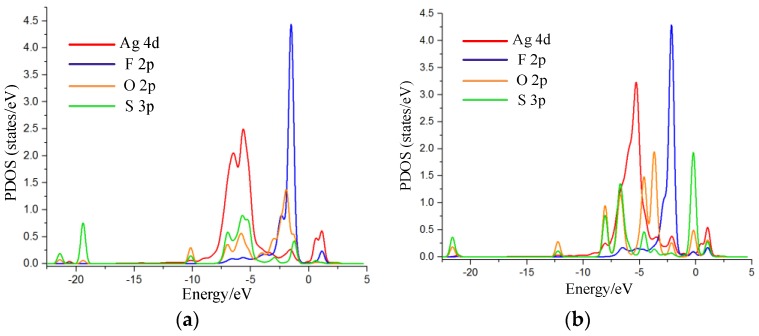

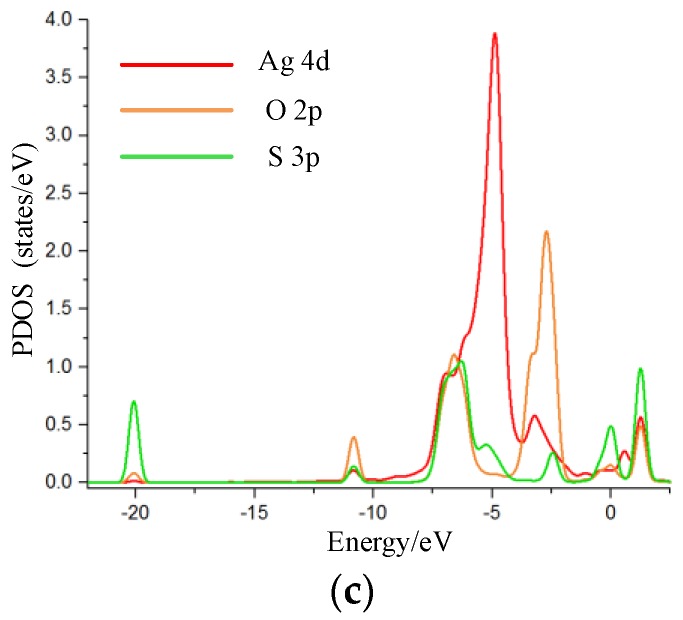
PDOS for Ag-graphene with and without single molecule adsorption; (**a**) PDOS for 4d of Ag atom, 3p of S atom, 2p of O atom, and 2p of F atom in SO_2_F_2_; (**b**) PDOS for 4d of Ag atom, 3p and 3d of S atom, and 2p of F atom in SOF_2_; and (**c**) PDOS for 3p of S atom and 2p of O in SO_2_.

DOS change was evident before and after SO_2_F_2_ adsorption by comparison, as shown in Figure 6a, where DOS decreased near the 0.8 eV conduction band and then increased in valence band range from −3 eV to −6 eV, which mainly led to an increase in the conductance of Ag-graphene/SO_2_F_2_ system. Figure 7a shows that PDOS peak near the −6 eV valence band causes DOS increase; because the 4d orbital of Ag atom hybridized with the 3p orbital of S atom and 2p orbital of O atom of SO_2_F_2_ in a certain filling rate degree, which bring a strong interaction between SO_2_F_2_ and decorated Ag.

DOS change before and after SOF_2_ only occurred below the Fermi level with a slight increase (see Figure 6b), suggesting that the conductivity of the system did not change, which was consistent with the conclusion of low adsorption energy discussed previously. More information from PDOS in Figure 7b reveals that a little energy overlap existed among atomic orbits. This indicated that the interaction between atoms was weak, which further verified that SOF_2_ adsorption on Ag-graphene belonged to physical adsorption.

Figure 6c shows DOS change during SO_2_ adsorption. Obvious change was not found before and after SO_2_ adsorption. However, the weak increase near valence bands of −3 eV and −6 eV was due to the net charge transfer at the effective overlapping part between the 4d orbital of the Ag atom, 2p orbital of the S atom, and 2p orbital of the O atom in PDOS (see Figure 7c). As a result, the effective charge number increased on a macroscopic scale after SO_2_ molecule adsorption, as did the conductance of Ag-graphene/SO_2_.

**Figure 8:**
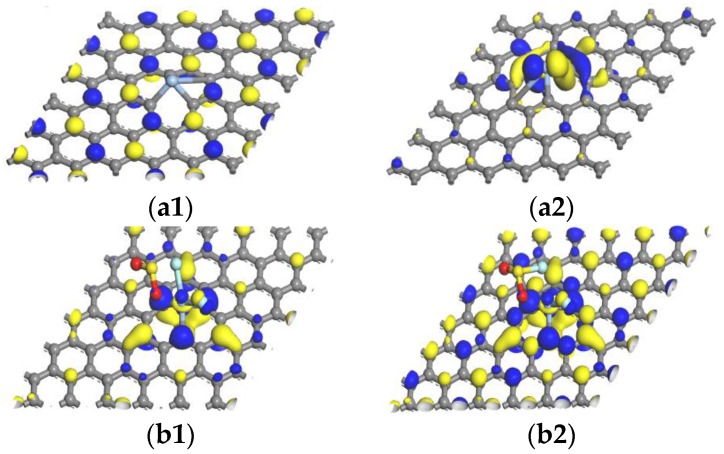

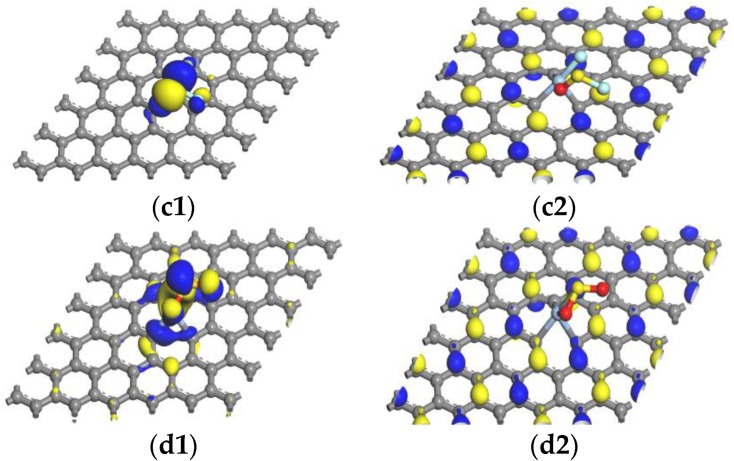
HOMO and LUMO: (**a1**,**a2**) Ag-graphene; (**b1**,**b2**) single SO_2_F_2_; (**c1**,**c2**) single SOF_2_; (**d1**,**d2**) single SO_2_.

We investigated the transition ability of electrons from the top of valence band to the bottom of conduction band and electronic structure through HOMO and LUMO. The calculation results of HOMO and LUMO of Ag-graphene structure before and after absorbing SF_6_ characteristic gas molecules (SO_2_F_2_, SO_2_ and SOF_2_) are shown in Table 4 and Figure 8.

Before absorbing gas molecules, the HOMO and LUMO of Ag-graphene were mainly distributed at the Ag-decorated site and its opposite site in Figure 8a1,a2, and the energy gap of Ag-graphene was 0.272 eV (see Table 4). The energy decline of HOMO and LUMO was due to the adsorption process, but its HOMO–LUMO energy gap increased in the system. When SO_2_F_2_ was adsorbed on Ag-graphene, the energy gap of Ag-graphene/SO_2_F_2_ increased to 0.761 eV, which indicated that the SO_2_F_2_ adsorption greatly increased system conductivity. After SO_2_ was adsorbed, the energy gap of the system slightly increased to 0.359 eV, which supported that conductivity of the Ag-graphene/SO_2_ increased to a certain extent. However, SOF_2_ adsorption almost had no effect on changing the energy gap of the system. All cases were consistent with the conclusion obtained through DOS analysis above.

### 3.4. Adsorption of Double Gas Molecules on Ag-Graphene

**Figure 9:**
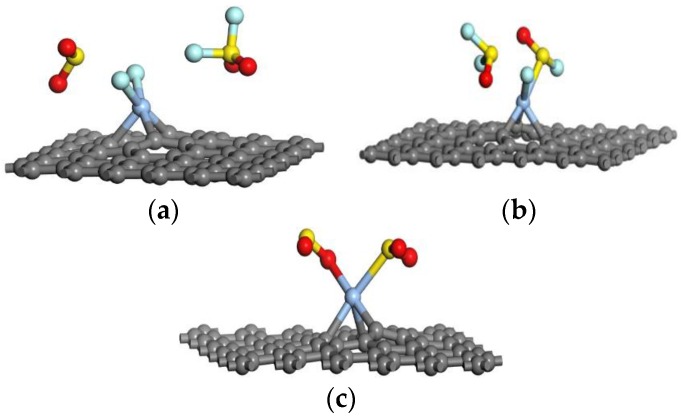
The most stable optimized geometries of double gas molecules interacting with Ag-graphene in (**a**) Ag-graphene/2SO_2_F_2_; (**b**) Ag-graphene/2SOF_2_; and (**c**) Ag-graphene/2SO_2_.

Before understanding the mechanism of gas molecules interaction with Ag-graphene, it was necessary to explore whether more gas molecules could be adsorbed onto the Ag-graphene surface. Thus, we constructed all kinds of configurations with two gas molecules (2SO_2_F_2_, 2SOF_2_, and 2SO_2_) adsorbed in favorable positions on Ag-graphene. Optimized systems (Ag-graphene/2SO_2_F_2_, Ag-graphene/2SOF_2_, and Ag-graphene/2SO_2_) are shown in Figure 9, and their related parameters are listed in Table 5.

As shown in Figure 9a, two F atoms break away from one SO_2_F_2_ and attached to the Ag atom, while another SO_2_F_2_ held its local position. In terms of bond length, only the S-F bond lengths of that SO_2_F_2_ were respectively extended to 2.241 Å and 3.612 Å with other bond lengths unchanged, indicating that these two S-F bonds were broken in the adsorption process. These results reveal that only one of the double SO_2_F_2_ gas molecules interacted with Ag-graphene. Based on Figure 5a and Figure 9a and Table 3, adsorption distances (d) are 2.013 Å and 2.004 Å, absorption energies (∆E_ads_) are −1.448 eV and −1.365 eV, and net charge transfers (Q) are −1.084 e and −1.113 e in Ag-Graphene/SO_2_F_2_ and Ag-Graphene/2SO_2_F_2_, respectively. By comparison, the adsorption process of double gas molecules is basically similar to that of the corresponding single gas molecule.

Similarly, when F and S atoms from the first SOF_2_ came close to Ag-Graphene with the S-F bond extended to 1.702 Å, the second SOF_2_ kept its molecule structure unchanged. As the adsorption distance (d) for single gas molecule of SOF_2_ was 0.051 Å shorter than that of a double gas molecule (see Table 3 and Table 5), ∆E_ads_ slightly decreased to −0.503 eV, and a total of 0.348 e electrons transferred from the gas molecule to the Ag-graphene. These demonstrated that only one of the double SOF_2_ gas molecules was adsorbed on the Ag-graphene.

However, an S atom from one SO_2_ and an O atom from another SO_2_ approached the Ag atom, adsorbing in opposite directions. The bond lengths of S-O bonds, 1.546, 1.503, 1.498, and 1.493 Å, were received from the initial 1.480 Å during the adsorption of double SO_2_ gas molecules. In addition, the net charge transfer (Q) of Ag-Graphene/2SO_2_ (−0.701 eV) were twice as much as that of Ag-Graphene/SO_2_ (−0.350 e) with considerable adsorption energy (∆E_ads_) of Ag-Graphene/2SO_2_ (−1.465 eV). Thus, new Ag–S and Ag–O bonds were formed, which led to electron enrichment of the Ag-graphene. These results reveal that more gas molecules were involved in interaction than Ag-Graphene/SO_2_.

**Figure 10:**
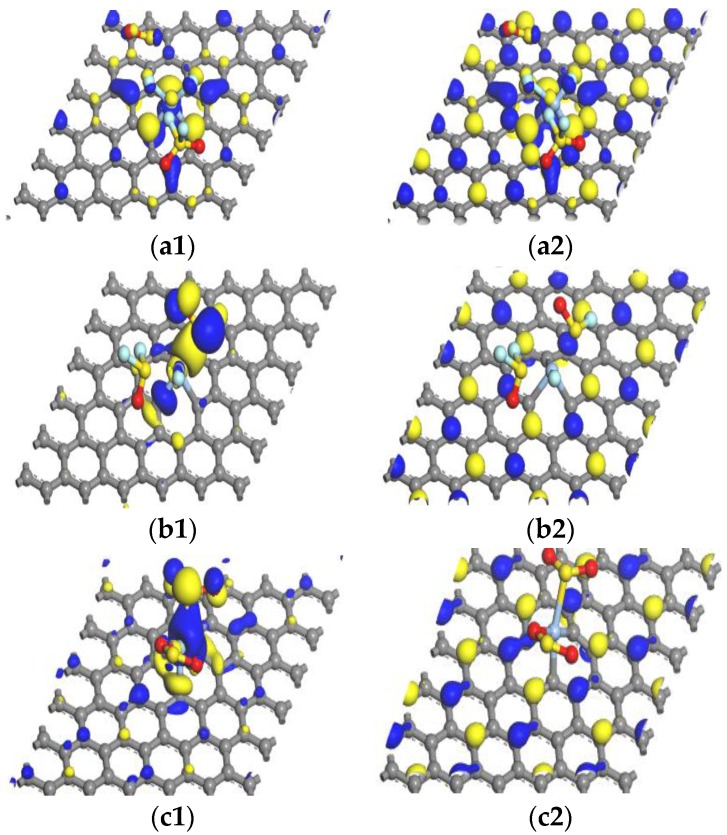
HOMO and LUMO: (**a1**, **a2**) double SO_2_F_2_; (**b1**, **b2**) double SOF_2_; (**c1**, **c2**) double SO_2_.

**Figure 11:**
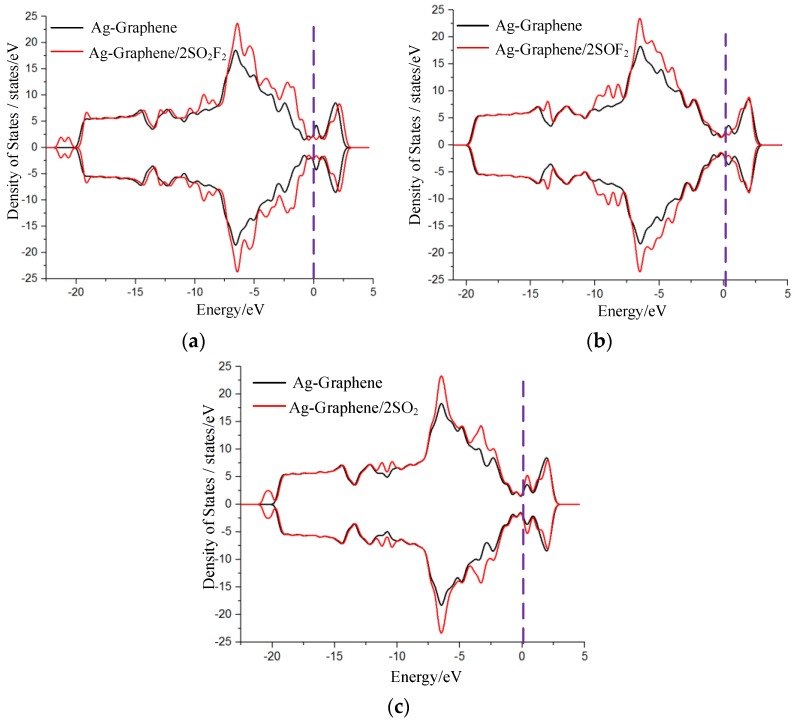
DOS for Ag-graphene with and without double molecule adsorption in (**a**) SO_2_F_2_; (**b**) SOF_2_; and (**c**) SO_2_.

As shown in Table 6, we calculated HOMO, LUMO, and energy gap after double gas molecules (2SO_2_F_2_, 2SOF_2_, and 2SO_2_) adsorbed on Ag-graphene. The adsorption results of double gas molecules are similar to that of single gas molecule adsorption; HOMO and LUMO energies declined after adsorbing gas molecules. When 2SO_2_F_2_ gas molecules were adsorbed on Ag-graphene, energy gap width increased to 1.050 eV, as shown in Table 6. The energy gap showed obvious changes compared to single gas molecule (−0.761), denoting that 2SO_2_F_2_ gas molecules made conductivity rise further. The HOMO gathered in SO_2_F, and all of LUMOs were located at Ag-graphene (see Figure 10b1,b2); and the HOMO-LUMO energy gap of Ag-graphene/2SOF_2_ was 0.282 eV, which was slightly higher compared with the intrinsic Ag-graphene that limits the performance of Ag-graphene to detect SO_2_F in SF_6_ decomposition components. For 2SO_2_ gas molecules, HOMO was mainly concentrated around Ag atom and gas molecules, while LUMO mainly distributed at the C atoms of Ag-graphene, as shown in Figure 10b1,b2. The width of energy gap increased to 0.542 eV, as shown in Table 6, indicating that the adsorption energy of 2SO_2_ gas molecules increased system conductivity to a certain extent. Nevertheless, 2SOF_2_ adsorption did not have an impact on the energy gap of the system. All adsorption results of double gas molecules are consistent with the conclusion of the corresponding adsorption energy, DOS, and other parameters above. (see Table 5 and Figure 11).

## 4. Conclusions

In summary, we mainly investigated the adsorption properties and mechanism for decomposition into SF_6_ product gases (SO_2_F_2_, SOF_2_, and SO_2_) on Ag-graphene based on density functional theory. A series of parameters presenting the adsorption characteristics in the adsorption process were calculated, and the main conclusions of the adsorption process were obtained as follows:

(1) After calculating and comparing the binding energy of three different Ag atom adsorption positions on graphene, the T-site was the best decorated location for Ag atom.
(2) Comparing with weak gas molecule interaction with Int-graphene, the Ag atom significantly enhances the electrochemical reactivity of graphene, reflected in the change of conductivity during the adsorption process.
(3) For single gas molecule adsorption, SO_2_ and SO_2_F_2_ gas molecules were adsorbed on Ag-graphene with strong chemical adsorption effect, and the interaction strength decreased in following order: SO_2_F_2_ > SO_2_. Also, Ag-graphene physically interacted with SOF_2_. The information from DOS, HOMO, and LUMO supported the conclusion that the electrical resistivity of the system was reduced during the adsorption process.
(4) The adsorption results of double gas molecules were similar to those of single gas molecules, and the increase of the number of molecules did not change the type of interaction. The decorating Ag atom only provided one adsorption site, so that only one gas molecule could be adsorbed on Ag-graphene when 2SO_2_F_2_ or 2SOF_2_ were near Ag-graphene. However, the adsorption energy and net charge transfer were significantly improved compared with that of single gas molecule adsorption. Based on the system conductivity change, the electrical conductivity of the system would further increase with the increase of adsorbed molecules (2SO_2_F_2_ and 2SOF_2_).

Thus, Ag-graphene showed distinct interaction with SO_2_F_2_, a kind of decomposition product of SF_6_. Results of theoretical study played a guiding role in preparing a gas sensor with strong selectivity and high sensitivity in the experiment. Meanwhile, the conclusions are beneficial to realizing online monitoring and diagnosis of SF_6_-insulated equipment in engineering.

## Figures and Tables

**Table 1:** Doping formation energy of Ag-doped graphene surface.

Doping Sites	*H-site*	*B-site*	*T-site*
E_form_ (eV)	1.51	1.32	0.85

**Table 2:** Doping formation energy of an Ag-doped graphene surface.

System	d (Å)	∆E_ads_ (eV)	Bond Length (Å)	Q_t_ (e)
Int-graphene/SO_2_F_2_	3.486	−0.463	1.610 (S-F_2_), 1.612 (S-F_1_), 1.442 (O_2_-S), 1.442 (O_1_-S)	−0.004
Int-graphene/SOF_2_	3.531	−0.459	1.672 (S-F_1_), 1.702 (S-F_2_), 1.463 (O-S)	−0.002
Int-graphene/SO_2_	3.344	−0.298	1.481 (O_2_-S), 1.482 (O_1_-S)	−0.003

**Table 3:** Doping formation energy of the Ag-doped graphene surface.

System	d (Å)	∆E_ads_ (eV)	Bond Length (Å)	Q_t_ (e)
Ag-graphene/SO_2_F_2_	2.013	−1.448	3.942 (S-F_2_), 1.948 (S-F_1_), 1.488 (O_2_-S), 1.536 (O_1_-S)	−1.084
Ag-graphene/SOF_2_	2.029	−0.678	2.718 (S-F_1_), 1.683 (S-F_2_), 1.536 (O-S)	−0.153
Ag-graphene/SO_2_	2.210	−1.075	1.499 (O_2_-S), 1.582 (O_1_-S)	−0.350

**Table 4:** Calculated HOMO, LUMO and HOMO-LUMO energy gap.

System	E_HOMO_/eV	E_LUMO_/eV	E_g_/eV
Ag-graphene	−4.589	−4.317	0.272
Ag-graphene/SO_2_F_2_	−6.263	−5.502	0.761
Ag-graphene/SOF_2_	−5.230	−4.945	0.285
Ag-graphene/SO_2_	−5.271	−4.912	0.359

**Table 5:** SF_6_ decomposition products adsorption on Ag-graphene.

System	d (Å)	∆E_ads_ (eV)	Bond Length(Å)	Q_t_ (e)
Ag-graphene/SO_2_F_2_	2.004	−1.365	2.241 (S-F_1_), 3.612 (S-F_2_),	−1.113
			1.494 (O_1_-S), 1.487 (O_2_-S),	
			1.605 (S-F_3_), 1.606 (S-F_4_),	
			1.443 (O_3_-S), 1.444 (O_4_-S)	
Ag-graphene/SOF_2_	2.080	−0.503	1.702 (S-F_1_), 1.672 (S-F_2_),	−0.203
			1.514 (O-S), 1.671 (S-F_1_),	
			1.673 (S-F_2_), 1.460 (O-S)	
Ag-graphene/SO_2_	2.210	−1.465	1.546 (O_1_-S), 1.503 (O_2_-S),	−0.701
			1.498 (O_3_-S), 1.493 (O_4_-S)	

**Table 6:** Calculated HOMO, LUMO and HOMO-LUMO energy gap.

System	E_HOMO_/eV	E_LUMO_/eV	E_g_/eV
Ag-graphene	−4.589	−4.317	0.272
Ag-graphene/2SO_2_F_2_	−6.250	−5.200	1.050
Ag-graphene/2SOF_2_	−5.286	−4.903	0.282
Ag-graphene/2SO_2_	−5.644	−5.102	0.542
